# A rare clinic presentation of abdominal pain: rupture of splenic artery aneurysm: a case report

**DOI:** 10.1186/1757-1626-2-148

**Published:** 2009-10-05

**Authors:** Sezgin Sarikaya, Baki Ekci, Can Aktas, Asli Cetin, Didem Ay, Alp Demirag

**Affiliations:** 1 Yeditepe University Hospital, Department of Emergency Medicine, Devlet Yolu Ankara Cad 102/104, Kozyatağı/İstanbul, Turkey; 2 Yeditepe University Hospital, Department of General Surgery, Devlet Yolu Ankara Cad 102/104, Kozyatağı/İstanbul, Turkey

## Abstract

**Background:**

Splenic artery aneurysms (SAA) are uncommon but the most common visceral artery aneurysm. Splenic artery aneurysms are important to recognize because up to 25% may be complicated by rupture and the mortality rate after rupture is between 25% and 70%.

**Case report:**

We present a patient who have abdominal pain. Previously healthy 22-year-old female admitted to emergency department with abdominal pain. Her physical examination reveals only left upper quadrant tenderness. Suddenly she developed hypovolemic shock. On emergent laparotomy massive blood collection within peritoneal cavity and retroperitoneal space at the left upper quadrant was detected. The source of bleeding was evident as rupture of splenic artery aneurysm. Splenectomy was performed following the ligation of splenic artery proximal to lesion. On the tenth day she was discharged from the hospital with complete recovery.

**Conclusion:**

It is important to remember rupture of splenic artery aneurysm in patients with abdominal pain and hypovolemic shock status.

## Introduction

Splenic artery aneurysms (SAA) are uncommon but represent the most common visceral artery aneurysm [1]. Most patients present at the sixth decade of life. Splenic artery aneurysms occur predominantly in multiparous women [2]. The etiologic factors are in relation with angiodysplasia, portal hypertension, pregnancy and atherosclerosis [3]. Splenic artery aneurysms are important to recognize. Because up to 25% may be complicated by rupture and the mortality rate after rupture is between 25% and 70% [4]. We present a case who admitted to emergency department with rupture of splenic artery aneurysm.

## Case presentation

**Figure 1:**
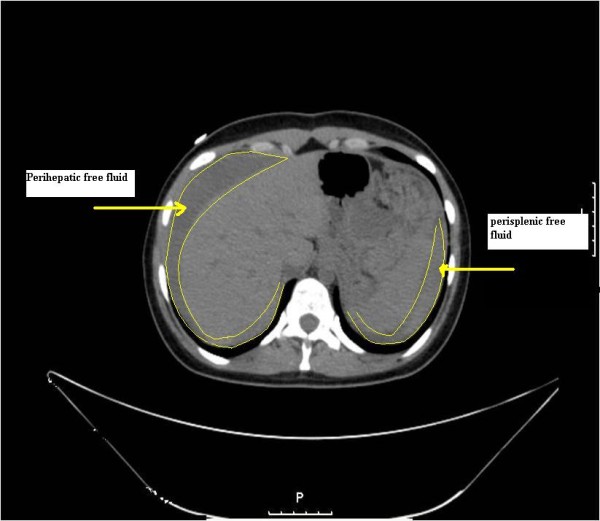
**Perihepatic and perisplenic free blood**.

A previously healthy 22-year-old female admitted to emergency department with abdominal pain which began two hours ago. There was no significant past history of medical illness. Her pulse was 70 beats/min, blood pressure 120/80 mm/Hg and body temperature was 36.5°C. On abdominal examination bowel sounds were normal, and she had left upper quadrant tenderness but neither defense nor rebound. Digital rectal examination was unremarkable. Hematological and laboratory values were within normal levels (hemoglobin 12.3 g/dL, hematocrit 38%, leukocyte count 7.91 × 10^3^/uL, platelet count 274 × 10^3^/uL). Urine analysis was unremarkable and βhCG was negative. An abdominal ultrasound was performed which revealed free fluid in Douglas pouch. Gynecological evaluation was requested and made a decision of observation in suspicion of tuboovarian cyst rupture. An abdominal computed tomography (CT) scan was planned on account of continuous pain and vomiting. Patient underwent computed tomography without delay. Since she was deteriorated, radiocontrast agent couldn't be given and survey was interrupted. After performing CT vital signs were evaluated as; blood pressure 60/30 mm/Hg, and heart rate 138/min. The new complete blood count resulted as hemoglobin 5,9 g/dL, and hematocrit 18, 9%, leukocyte count 8.360 × 10^3^/uL, platelet count 207 × 10^3^/uL. Rapid fluid resuscitation was started with isotonic saline solution and Gelatin polysuccinate 4%(Gelofusine). On the basis of abdominal CT; these images on the left of the spleen a heterogenous area with hypodense regions was observed. It was almost 100 mm in transverse diameter and probably in the form of free fluid collection. Diffuse free fluid was present in all quadrants of the abdomen (Figure 1). Then she was taken into emergency surgery. In the operation; massive blood collection within peritoneal cavity and retroperitoneal space at the left upper quadrant was detected. The source of bleeding was evident as rupture of splenic artery aneurysm which is located near hilus. Splenectomy is performed following the ligation of splenic artery proximal to lesion. The patient's postoperative course was uneventful and on the tenth day she was discharged from the hospital with complete recovery.

## Discussion

Splenic artery aneurysms (SAA) are uncommon and, accounts for about 60% of visceral artery aneurysms [1,2,5]. Autopsy studies show a prevalence of between 0.1% and 10.4%. The reported rate of rupture is between 3% and 9.6% with an associated mortality of 36 % [6]. Most aneurysms are small, saccular, and located at a bifurcation in middle or distal segment of the splenic artery.

In this patient we found a small saccular aneurysm at the distal part of splenic artery [7,8]. The pathogenesis of SAAs is not fully understood. It can occur in atherosclerosis, mycotic infection, blunt abdominal trauma, essential hypertension, portal hypertension, chronic pancreatitis, diabetes, polyarteritis nodosa, arterial dysplasia, pregnancy and liver transplant patients [3,9,10]. The presented case had none of the above mentioned factors. Pathological examination was not giving information.

Patients are often asymptomatic, and only 20% have symptoms such as vague left upper quadrant or epigastric discomfort or back pain, and most splenic artery aneurysms are detected incidentally during diagnostic imaging performed for other indications [6-8,10]. In our case, the patient was presented to the emergency department with left upper quadrant abdominal pain. When the patient performed abdominal tomography, her hemodynamic stability was changed. And then, emergency laparotomy was done.

Rupture is the most fatal clinical presentation of the splenic artery aneurysm. Life-threatening rupture results in severe abdominal pain or even hypovolemic shock. The highest incidence of rupture is in young pregnant women [5].

The treatment of SAA depends on its locations over the splenic artery. The treatment for splenic artery aneurysm has been bipolar surgical ligation of the splenic artery, ligation of the aneurysm, or aneurysmectomy with or without splenectomy depending on the aneurysm location. If the aneurysm is located in the distal portion of the splenic artery, the standard treatment is aneurysmectomy with splenectomy [5,8,10].

In a recent case, median laparotomy was done. Massive blood collection within peritoneal cavity and retroperitoneal space prominently at the left upper quadrant was detected. At retroperitoneal exploration, the source of bleeding was found near splenic hilus. Splenectomy was performed following the ligation of splenic artery proximal to lesion.

On the other hand, if aneurysm can be found incidentally, it also may be treated with percutaneous interventional techniques such as transcatheter embolization, placement of a covered stent-graft to exclude the aneurysm, or percutaneous injection of coils or thrombin [5-8,10].

Splenic artery aneurysm rupture, although rare, may become a relevant differential diagnosis of intraperitoneal hemorrhage and sudden death, respectively. When the patient come to emergency department with epigastric and abdominal pain and hypovolemic shock status, vessel aneurysm rupture or rupture of splenic artery aneurysm remember.

## Abbreviations

SAA: Splenic artery aneurysms; CT: computed tomography.

## Consent

Written informed consent was obtained from the patient for publication of this case report and accompanying images. A copy of the written consent is available for review by the Editor-in-Chief of this journal.

## Competing interests

The authors declare that they have no competing interests.

## Authors' contributions

SS analyzed and interpreted the patient data. BE, and CA was the major contributor in writing. AC and DA helped in searching the literature. BE and AD involved in treatment management. AD involved in checking all the course.
